# Smartphone Camera Identification from Low-Mid Frequency DCT Coefficients of Dark Images

**DOI:** 10.3390/e24081158

**Published:** 2022-08-19

**Authors:** Adriana Berdich, Bogdan Groza

**Affiliations:** Faculty of Automatics and Computers, Politehnica University of Timisoara, 300223 Timisoara, Romania

**Keywords:** smartphone, camera sensor, fingerprinting, DSNU, AC coefficients, machine learning

## Abstract

Camera sensor identification can have numerous forensics and authentication applications. In this work, we follow an identification methodology for smartphone camera sensors using properties of the Dark Signal Nonuniformity (DSNU) in the collected images. This requires taking dark pictures, which the users can easily do by keeping the phone against their palm, and has already been proposed by various works. From such pictures, we extract low and mid frequency AC coefficients from the DCT (Discrete Cosine Transform) and classify the data with the help of machine learning techniques. Traditional algorithms such as KNN (K-Nearest Neighbor) give reasonable results in the classification, but we obtain the best results with a wide neural network, which, despite its simplicity, surpassed even a more complex network architecture that we tried. Our analysis showed that the blue channel provided the best separation, which is in contrast to previous works that have recommended the green channel for its higher encoding power.

## 1. Introduction and Motivation

Artificial intelligence and the Internet of Things (IoT) are currently in fast evolution and interaction. With more than 20 billion devices connected to the Internet and huge amounts of information that comes from such devices, the need to automatically check the source of information is a stringent demand. Currently, all mobile devices are equipped with digital cameras, and using images collected by cameras is a convenient method for identifying smartphones.

**Figure 1:**
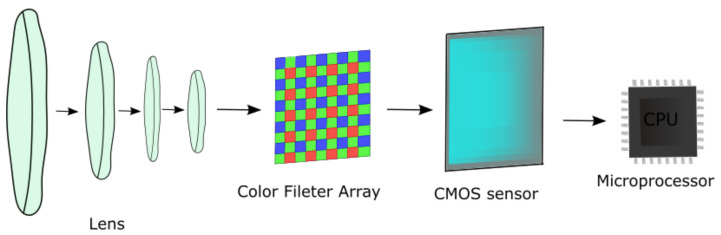
Suggestive depiction of smartphone camera.

The most important component of the digital camera is the sensor. The sensor converts the captured light into electric signal to produce black, white, and gray pixels. The colors are obtained by applying a color filter array (CFA). The most used CFA is the Bayer Filter Array, which alternates the three colors red, green, and blue. Since our eyes are more sensitive to green, in the Bayer Filter Array, half of the array contains green filters, and the rest is split between red and blue. The digital cameras can have two types of sensors: a CCD (Charge-Coupled Device) sensor or a CMOS (Complementary Metal-Oxide Semiconductor) sensor. Since CMOS sensors are low power and less expensive than CCD sensors [1], the majority of smartphones are equipped with CMOS sensors, a reason for which we focus on such sensors alone in this work. In Figure 1, we depict the main components of the smartphone camera. The light passes through a lens and then goes through a color filter. The CMOS sensor converts the light corresponding to the three colors, red, green, and blue, into digital information.

A specific use case is the design of Physical Unclonable Functions (PUF), which, for a set of inputs (seen as challenges), give a device-specific response based on unique and unpredictable circuit variations that result from the manufacturing process. These variations account for unique sensor characteristics given by physical, chemical, and geometrical imperfections in sensors. The concept of circuit identification based on unique information from the randomness of the manufacturing process was introduced two decades ago [2], and the PUFs emerged slightly later [3]. Since then, many PUFs were proposed. Based on the number of the challenge–response pairs (CRPs), there are two types of PUFs: weak PUF (have a limited number of CRPs), e.g., memory-based PUFs, and strong PUF (huge number of CRPs). The PUFs are commonly used in device authentication and device/sensor fingerprinting systems.

**Figure 2:**
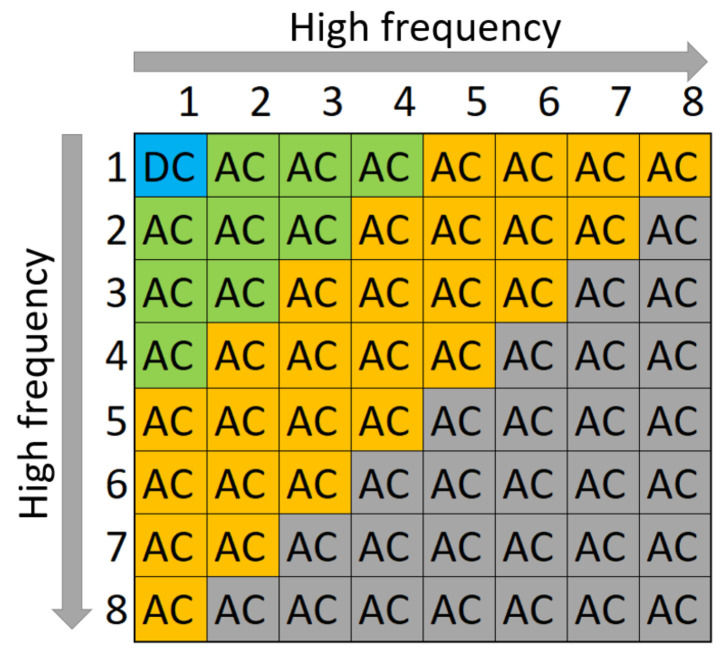
DCT coefficients.

Returning to camera sensors, fixed-pattern noise (FPN) is a constant variation in pixels recognizable as the same pattern of darker and brighter pixels in images that are taken in uniform illumination conditions. The FPN can be defined as $\mathrm{FPN}=X-X_{\mathrm{fil}}$, where *X* is the original image and $X_{\mathrm{fil}}$ is the filtered image. The noise extracted by this procedure depends on various manufacturing imperfections that are unique to each sensor. Furthermore, this noise can be used to generate a PUF since it contains characteristics that are unique to a device and can be further used as inputs to a cryptographically secure function as presented in various works like [4,5,6]. There are two types of FPNs: (i) dark signal nonuniformity (DSNU), which accounts for variations in pixels offsets under no illumination conditions (dark frame), and (ii) photo response nonuniformity (PRNU), which is a variation between pixels under illumination conditions (light frames). In this work, we focus on DSNU alone, which gives more accuracy and is no less practical to use. Discrete Cosine Transform (DCT) is a method to transform image pixels into the frequency domain from the spatial domain. DCT is commonly used in the JPEG compression algorithm for images. In JPEG compression, the two-dimensional Discrete Cosine Transform (2-DCT) is applied on × nonoverleaping blocks of the image. For each 8 × 8 block, 64 DCT coefficients are obtained, from which the first coefficient, from the upper left corner, is the DC coefficient and the remaining 63 are the AC coefficients [7]. In Figure 2, we depict the DC coefficient in blue, low-frequency AC coefficients in green, mid-frequency AC coefficients in orange, and finally, the high-frequency AC coefficients in gray (these correspond to the DCT of an × image block; this block size is specified for encoding in the ISO/IEC 10918-1:1994 standard for digital compression and coding of digital images [7]). Since high-frequency coefficients are sensitive to distortions, they are influenced by JPEG compression, and in this work, we use only the low and mid-frequency AC coefficients. Additionally, the human eye is more sensitive to low frequencies and less sensitive to high frequencies.

To analyze such frequency domain representations from data collected on six identical CMOS sensors, we use several traditional machine learning algorithms, i.e., Ensemble-Subspace Discriminant (ENS), Nearest Neighbor (KNN), Support Vector Machines (SVM), Naive Bayes (NB), Linear Discriminant (LD), and a wide neural network (WNN). For a more accurate image on related approaches, in Table 1, we summarize the results from some works that use machine learning and deep learning in particular for camera identification. The results obtained by other works are comparable with our results obtained with WNN for 1000 samples. Note that these results are not on identical datasets. Most of the works use public datasets such as Vision [8] that contain images in the original format as well as in the format in which they are stored on social networks. Other datasets such as Dresden [9], ISA UNICAMP [10], and Flickr (https://www.flickr.com/ accessed on 25 March 2022) contain various indoor and outdoor scenes that have been used in various works. The highest accuracy is achieved by [11], and it is for the case of 74 digital cameras (not smartphone cameras) from a public dataset [9] from 2010 that contains a maximum of 3 identical devices. While this table offers a good image on the overall accuracy, the results cannot be immediately compared since distinct images, subject to availability, were used. In our work, we choose to focus on 6 identical camera sensors that are coupled to the same phone since a scenario with identical sensors seems to offer the most challenging circumstances.

The rest of the work is organized as follows. In Section 2, we analyze some related works. Section 3 depicts the devices, tools, experimental scenario, and gives a overview of the concept. In Section 4, we validate our method using distinct machine learning algorithms. Section 5 holds the conclusion of our work.

## 2. Related Work

There is a rich literature available on the analysis of PUFs from CMOS sensors. A PUF that exploits the intrinsic randomness of oxide breakdown in CMOS sensors transistors using 40 nm CMOS chips is proposed in [19]. The authors in [20] propose a PUF from CMOS image sensors based on pixel variations. A design for process-sensitive optical nanostructures in CMOS sensors to create an optical PUF based on resonant photonic crystals for 65-nm CMOS chips is proposed in [21]. The authors in [22] proposed a tamper resistant hash based on device and camera properties that serves as a PUF to identify the device and detect manipulations. The dynamic vision sensor (DVS) technology is used in [23] with an implementation realized on 1.8 V, 180 nm CMOS technology.

Since some camera models include their own SRAM, the SRAM was used to generate a PUF for IoT security cameras in [24]. Six different types of memory-based PUFs, i.e., SRAM PUF, Latch PUF, D flip-flop PUF, Buskeeper PUF, and two delay-based PUFs, Arbiter PUF and a Ring Oscillator PUF, are analyzed in [25] using 192 chips with 65 nm low-power CMOS sensors. More recently, the use of biological PUFs has been suggested based on an example that exploits the randomness in T-cell populations [26].

A user authentication method based on smartphone camera identification is proposed in [27]. For this, a PUF is extracted from the high-frequencies components of PRNU estimated from RAW images. The idea to use PRNUs for source camera identification is not new. Digital camera identification based on sensors pattern noise was proposed as early as 2006 in [28]. The camera identification algorithm consists of computing the correlation between the residual noise and a reference pattern noise, which results as a mean value of the residual noise from multiple images. In the recent years, several papers have been published that explore different techniques for source camera identification based on PRNU. The PRNU estimated with the Maximum Likelihood principle is discussed in [29].

A computationally efficient method for source camera identification and verification using PRNU is described in [30]; the PRNU is extracted based on joint edge preserving filtering. The authors of [31] discuss camera source classification based on the correlation between the reference PRNU pattern and the PRNU pattern of the image extracted after denoising the image. PRNU fingerpints are also used in [32,33] for camera identification.

Source camera identification based on PRNU does not normally work on HDR images because these images usually result from combining subsequent shots from different exposures, which increases the camera offset range. The authors in [34] proposed a method for camera identification for HDR images using PRNU based on reversal spatial transformations. Source camera clustering based on PRNU, for a criminal case dataset, is presented in [35]. Device identification based on camera sensor is proposed in [17], where noise print information is used to support PRNU for camera source identification. Three classification methods were used: SVM, Likelihood ratio test, and Fisher’s linear discriminant. The authors in [36] proposed a new method for source camera identification verification based on PRNU. An improved Locally Adaptive Discrete Cosine Transform (LADCT) filter is used to remove the noise, and for estimations, the Weighted Averaging (WA) is proposed. A solution for video source identification based on comparison of the green channel photoresponse nonuniformity (G-PRNU) extracted from frames of the video with the reference G-PRNU extracted from a pool of images is discussed in [37]. Bilinear interpolation is applied to resize the frames and images to 256 × 256 pixels. The PRNU is amplified using a sharpening technique, i.e., Unsharp Masking, in [38]. The PRNU was extracted from the green channel and a wavelet-based filter was used for denoising. As the camera fingerprint extraction based on PRNU may be expensive, in terms of computational time for a large number of images that need to be denoised, the authors of [39] proposed a method for camera fingerprinting based on Spatial Domain Averaged (SDA) frames, which consists of computing the average of the images before denoising. This method improves the computational time for camera fingerprint extraction by 50 times compared with other methods based on PRNU. A technique for source camera identification based on the SPN extracted from images using a method based on dual tree complex wavelet transform is proposed in [40]. The authors in [5,6] discuss a method of smartphone CMOS camera sensor fingerprint generation for sensor level authentication based on PUF extracted from DSNU of FPN (fixed-pattern noise). A mobile devices CMOS image sensors PUF based on DSNU of FPN used in an authentication process is analyzed in [4]. A method to improve the performance of source camera identification by removing the suppressing peaks in the Fourier domain and removing the low-frequency defects of the reference sensor pattern noise (SPN) in DCT domain is discussed in [41]. The low-frequency defects are produced by reflection of the light on optical surface and dust particles. Camera model identification from JPEG images based on DCT coefficients is analyzed in [42]. The authors analyze the camera model identification in optimal conditions, with likelihood ratio tests, having known model parameters. Two Generalized Likelihood Ratio Tests are presented for practical contexts, when the model parameters are unknown.

There are also many papers addressing source identification, from camera data, with the help of machine learning and deep learning algorithms in particular. Source camera identification using machine learning algorithms on the features extracted from DCT and Random Forests with AdaBoost is discussed in [13]. The camera brand identification, camera model identification, and device-level identification using original and manipulated images using multitask learning is analyzed in [43]. The feature coupling, which maps the original features of the images to a coupled feature representation, is used to obtain a probability representation by training the multiclass SVM classifier in [14]. The probability representation is converted into the coupled probability representation, which is used to predict the image source. The authors in [44] discuss a method for source camera brand identification using machine learning. Ensemble classifier based on demosaicing residual features is used in [45].

Camera model identification using CNN and transfer learning is discussed in [46]. Three classifiers were used on a pretrained model: SVM, Random Forests, and the Logistic Regression Model. Camera model identification using a multiclassifier based on CNN is analyzed in [11]. By using the majority voting, the solution achieves an average accuracy nearly to 100%; it was also validated for JPEG compression and noise addition. The authors in [47] discuss a camera identification method for postprocessed images based on extended Bayar CNN. Commonly used image manipulation techniques such as compression, scaling, and contract enhancement were analyzed. Source camera identification of Facebook images based on deep learning ResNet50 network is discussed in [15]. The Res2Net image model is used in [48]. The authors in [49] use CNN and RemNet. Source camera identification based on content-adaptive fusion network is discussed in [50]. A multiscale content-independent feature fusion network (MCIFFN) is proposed in [51]. A significant number of works employ convolutional neural networks (CNN) for device identification, e.g., [18,52,53,54]. Source camera identification by brand, model, and device using coupling multitask training based on CNN is discussed in [55]. Due to low performance for camera model classification, the authors propose an auxiliary classifier used on the local neighborhood differences for the camera lens. This auxiliary classifier is focused only on camera model reclassification. The camera model identification using CNN based on the preprocessing images using a high-pass filter is discussed in [12]. The authors demonstrate that the CNN network is good for a smaller complexity comparison with GoogleNet, and the discussed CNN model is improved in comparison with AlexNet.

Other methods for source camera identification may be also worth mentioning. The authors in [56] discuss source camera identification based on image texture features. They extract the Local Binary Pattern (LBP) and Local Phase Quantization features from the residual noise of the image. The extracted features are used by Multiclass Lib-SVN classifiers to identify the source camera. The I-Vector, usually employed in speech processing, is used for camera identification in [57]. Distinct feature extraction algorithms and classifiers for camera model identification are compared in [16]. The removal of the least significant bits and Gaussian blurring is discussed in [58].

In addition to this, the recent literature has considered smartphone identification using distinct sensors, e.g., microphone [59,60], loudspeaker [61,62,63], gyroscope [64], battery consumption [65], accelerometers [66,67], etc.

## 3. Setup and Methodology

**Figure 3:**
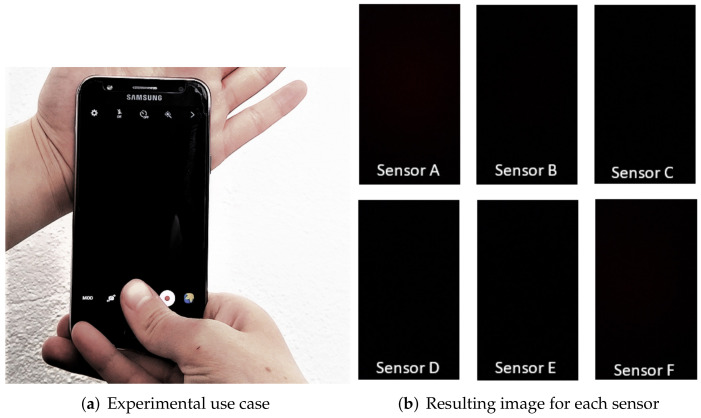
Experimental use case (**a)** and one image for each camera sensor (**b**).

In this section, we give a brief overview of the setup and methodology. First, Figure 3a shows the experimental use case: a user taking a dark picture by holding the phone against their palm to fingerprint the sensor. In Figure 3b, we show an example of 6 dark images captured with the 6 cameras. This movement is simple, and users can collect such data without much effort. We acquired 50 dark images with each camera by this procedure. The experiments were performed at room temperature, i.e., around 22 ∘C elsius.

**Figure 4:**
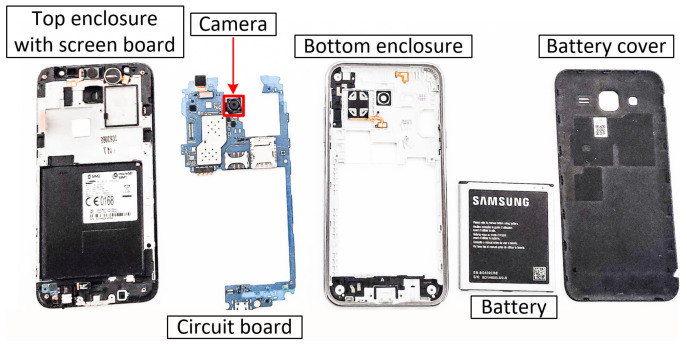
Samsung Galaxy J5 dismantled to replace the camera.

**Figure 5:**
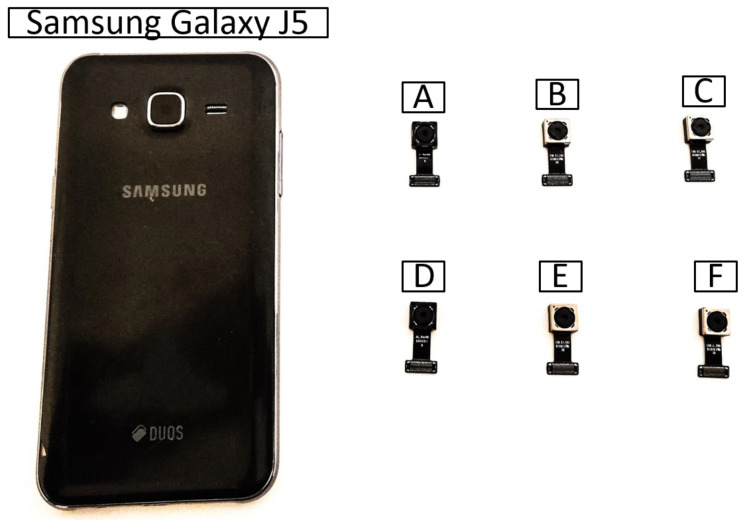
Samsung Galaxy J5 along with the six dismantled cameras.

In our fingerprinting scenario, we use a Samsung Galaxy J5 smartphone with 13 MP sensors, f1.9, 28 mm (wide) lens, AF capable camera. Figure 4 shows the dismantled Samsung Galaxy J5 for camera replacement. To the original smartphone, we add six identical camera circuits (these are connected to the same Samsung Galaxy J5 smartphone) in order to avoid imperfections due to the rest of the electronics inside the smartphone and obtain an accurate measurement for the imperfections in each sensor. Indeed, using distinct smartphones will also contribute to such differences. Our intention was to get an accurate measurement for the imperfections in each sensor. In Figure 5, we show the Samsung Galaxy J5 along with the six cameras. For analyzing and processing the images, we used MATLAB R2020b. The analysis was conducted on a notebook with an Intel(R) Core(TM) i7-6700 processor at 3.40 GHz with 32 GB RAM.

**Figure 6:**
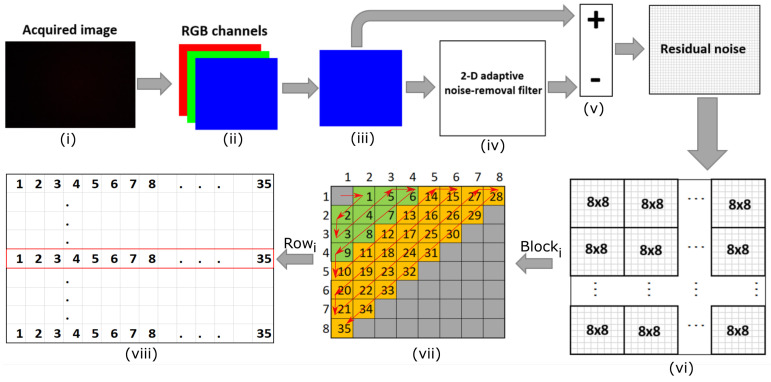
Fingerprint extraction process: from image acquisition to AC coefficients.

In our investigations, we analyzed portions extracted from the DCT applied over the entire picture, avoiding cropping or resizing, which can influence the fingerprint. We only considered the blue channel because the results were better, as shown in a future paragraph. Additionally, by using a single channel, we improved the computational time. We used a 2-D adaptive noise removal filter, i.e., the *wiener2* filter from MATLAB, in order to process the original image. This filter estimates the variance and local mean around each pixel with 10 × 10 local neighborhoods. To extract the pixel variations, we compute the residual noise as the difference between the original image and the filtered image. We split the residual noise in 8 × 8 nonoverleaping blocks. As we also mentioned in the introduction, for each block, we compute the 2-D DCT and we extract the low and mid frequency AC coefficients. Using the zig-zag sequence, from each 8 × 8 block, we obtain an array with 35 elements which are concatenated to obtain the fingerprint. Figure 6 depicts this fingerprint extraction process, which consists in the following seven steps: (i) image acquisition, (ii) splitting the image into RGB channels, (iii) extracting the blue channel, (iv) applying the Wiener2 filter to the blue channel, (v) computing the residual noise as the difference between the blue channel image and the filtered image, (vi) splitting the residual noise into × blocks, (vii) applying 2-D DTC on each 8 × 8 block, and (vii) extracting the low and mid AC coefficients.

*Entropy analysis.* To begin with, we outline the entropy of our data at the beginning and the end of the processing steps from Figure 6. As a metric, we use both the Shannon entropy and the minimum entropy [68], the latter being a more useful security metric (for applications that intend to use CMOS data as a PUF for authentication) in case of an adversary that simply tries to guess the data produced by the sensor by using the most likely value of the coefficients. The former is computed using the relation $\sum_{i=0.255}-p_{i}\log p_{i}$ and the latter as $-\log\max(p_{i})$ where $p_{i},i=0.255$ is the probability of occurrence for each byte in the array (the array represents either the RGB bytes in the original image or the bytes of the AC coefficients).

**Figure 7:**
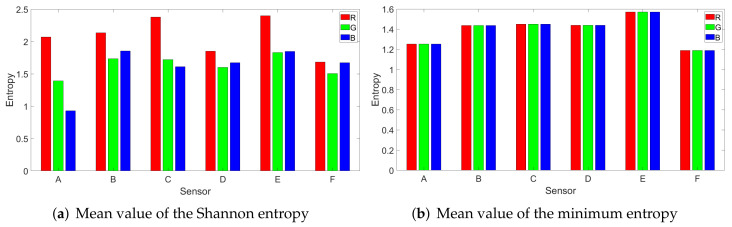
Shannon (**a**) and minimum (**b**) entropy computed on the red, green and blue channels form the image.

In Figure 7, we depict the two entropies, i.e., the Shannon (a) and minimum (b) entropy, as computed on the red, green, and blue channels of the image (without any processing). The red channel has slightly higher entropy values than the green and blue channels, while the blue channel has the lowest entropy, which strengthens the reason to choose it in the classification process, as it will give more stable results. For the red channel, the mean of the Shannon entropy is 2.0855 and its median is 2.1133. In case of the green channel, the mean is 1.6310 and the median is 1.6354, while for the the blue channel, the mean is 1.5982 and the median is 1.6024. For the minimum entropy, the values on the three channels are nearly identical, suggesting an equal minimum security level.

**Figure 8:**
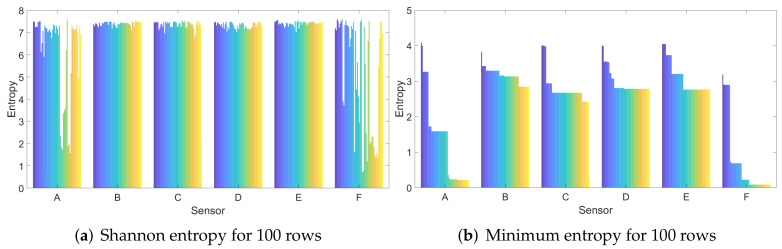
Shannon (**a**) and minimum (**b**) entropy for 100 randomly selected rows.

We now compute the values for the Shannon and minimum entropy on the extracted AC coefficients. As expected, the values are higher than previously, generally reaching around 7 bits for each byte in case of the Shannon entropy. This is expected since the image is essentially squeezed into these AC coefficients that represent the color changes. In Figure 8, we depict the Shannon and the minimum entropy per coefficient in case of 100 randomly selected rows. The matrix on which the entropy was computed has 2800 elements. For sensors A and F, the entropy occasionally drops, likely due to environmental factors, as it is hard to take identical dark images when pressing the phone against the palm. However, the minimum entropy is still generally in the range of 2–3 bits for each byte from the coefficients, twice than in the case of the unprocessed images, and for a matrix of 2800 elements, the security level is sufficiently high.

**Figure 9:**
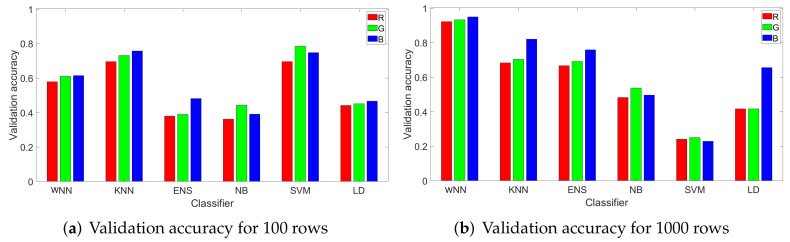
Validation accuracy for 100 (**a**) and 1000 (**b**) randomly selected rows for all classifiers and all channels.

*Channel selection.* While other works have generally used the green channel for camera identification, we choose the blue channel since our preliminary analysis suggested that this channel gives better results. To support this hypothesis, in Figure 9, we depict as bar charts the validation accuracy for 100 and 1000 randomly selected rows for all classifiers and all three channels. We mark each channel with its corresponding color: red, green, and blue. We used 80% of data for training and the rest of 20% for testing. As it can be easily observed, the blue channel has the best accuracy with NN, KNN, ENS, and LD, while for NB and SVM, the green channel has the better accuracy. Still, NB and SVM give the worst results for all channels on 1000 samples, which deems them unsuitable as classification algorithms for this purpose.

**Figure 10:**
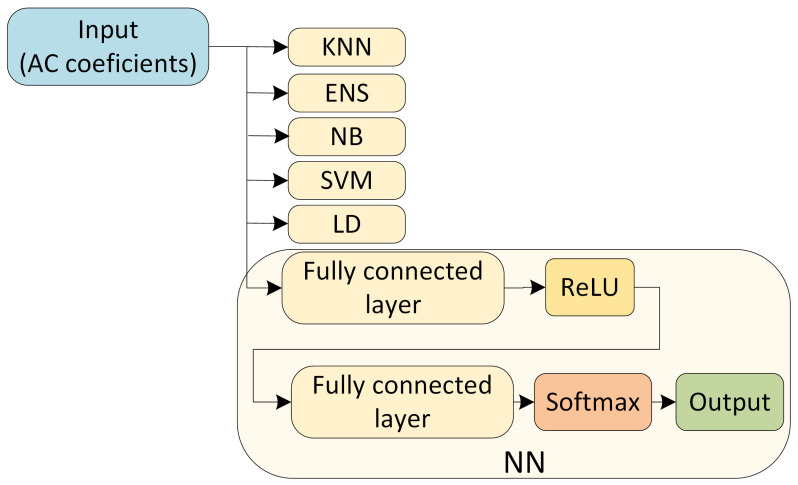
The AC coefficients as input for the multilayer fully connected neural network and the classifiers.

After processing the images, for each image, we obtain a bidimensional array with 149,640 rows and 35 columns. The 149,640 rows correspond to each of the 8 × 8 matrices obtained for each image, while 35 is the number of extracted AC coefficients. Due to the large size of the output array obtained after processing, the classification based on the full array is not practical due to two factors: prediction time and memory requirements (out of bounds errors may result from several classifiers). To circumvents such problems, we select samples of 100 or 1000 rows and use them for each image and device. The 100 rows (or 1000 in the second case) were selected at random from the 149,640 rows, but the selection was kept identical for all images in the experiment. As a result, to perform the classification, each image is translated into a bidimensional array of 100 or 1000 rows and 35 columns. We also tried to use 10,000 rows, but the improvements in the accuracy of the results were not great while the classification time increased from some classifiers, and for others, such as LD, SVM, and NB, we received an out-of-memory error due to the large size of the dataset. Another attempt was to use the top left corner of each image and take 100 or 1000 element matrices from there, but the results were slightly worse. The previously mentioned random selection of the matrices seemed to give the best results. For all the classification algorithms that we used, i.e., WNN, KNN, ENS, NB, SVM, and LD, we use as input 100 (or 1000) rows and 35 columns with the low and mid AC coefficients obtained after applying the 2-D DCT on the × blocks from the residual noise that we extracted from the images. In Figure 10, we depict the inputs of the classification algorithms.

## 4. CMOS Sensor Identification with Machine Learning Algorithms

In this section, we discuss the CMOS sensor identification using several classifiers, i.e., linear discriminant (LD), Support Vector Machine (SVM), Naive Bayes (NB), Ensemble-Subspace Discriminant (ENS), Nearest Neighbor (KNN), and a multilayer fully connected neural network (NN).

### 4.1. Selected Classifiers

We briefly describe the classifiers that we use for CMOS sensor identification providing details on some of the parameters. The discussion that follows is mostly based on the arguments provided by the MATLAB documentation from [69].

#### 4.1.1. Wide Neural Network Structure (WNN)

The wide Neural Network (WNN) that we use contains an input layer, followed by a fully connected layer with 100 neurons. This is a simple neural network available as default, but it proves to be surprisingly effective for our dataset. Much to our surprise, by using a basic convolutional neural network (CNN), the results were slightly worse on our dataset. For the activation function of the fully connected layers, we use a rectified linear unit (ReLU), which performs a threshold operation to remove the negative values as follows:

$$f\left(x\right)=\left\{\begin{array}{cc} x, & x\geq 0\\ 0, & x<0 \end{array}\right.$$

Then, we use a final fully connected layer with 6 outputs that correspond to the 6 sensors that we use. For the activation of the final fully connected layer, we use a Softmax function, which normalizes each input into a probability distribution. In Figure 10, we show the architecture of the multilayer fully connected neural network that we used, i.e., the WNN.

#### 4.1.2. Fine KNN (KNN)

There are six Nearest Neighbor classifiers available in MATLAB, i.e., Fine KNN, Medium KNN, Coarse KNN, Cosine KNN, Cubic KNN, and Weighted KNN. The prediction speed is medium for all types of KNN, except for the cubic KNN, which has a lower prediction speed. The KNN classifiers have average memory requirements, and they are slightly hard to interpret. In this work, we use the Fine KNN classifier because it seemed to be more compatible with our datasets, i.e., it gave the best results compared to the others. We use 10 neighbors and the Euclidean distance as a metric, with equal distance weight, and we also normalize the data.

#### 4.1.3. Ensemble—Subspace Discriminant (Ensemble)

There are several ensemble classifiers algorithms, e.g., boosted trees, which includes AdaBoost learners, Bagged Trees with includes Random forest, Subspace Discriminant, Subspace KNN, RUSBoost Trees, and GentleBoost. The prediction speed for ensemble algorithm varies depending on classier type from fast to average. Additionally, the memory usage can be low, medium, or high depending on classier type. In this work, we use the Subspace Discriminant classifier because it proved to perform better on our datasets. The prediction speed and memory usage are average for this classifier. We obtain the best performance for this classifier using 30 learners and a subspace dimension fixed to 1750, i.e., for each learner, we used 1750 predictors.

#### 4.1.4. Naive Bayes (NB)

Naive Bayes includes two types of classifiers Gaussian Naive Bayes and Kernel Naive Bayes. In this work, we use the Kernel Naive Bayes. This classifier is recommend for multiclass classification and is easy to interpret. As the name suggests, this classification algorithm is based on Bayes’s theorem.

#### 4.1.5. Linear SVM (SVM)

Support Vector Machines can be used to train binary or multiclass models. The prediction speed and memory usage depends on class type, i.e., for binary classes, the prediction speed is fast and memory usage is medium for all classifier types, while for multiclass, large amounts of memory are used and the prediction speed is from medium to low depending on classifier type. There are 6 classifier types, i.e., Linear SVM, Quadratic SVM, Cubic SVM, Fine Gaussian SVM, Medium Gaussian SVM, and Coarse Gaussian SVM. In this work, we use the Linear SVM classifier because it proved to be more suitable for our datasets. We use a linear kernel function, automatic kernel scale, one box constraint level, and one-vs.-one multiclass method.

#### 4.1.6. Linear Discriminant (LD)

Discriminant analysis is a classification algorithm with a fast prediction speed and high accuracy that is easy to interpret. Based on the type of Gaussian distribution that is used, there are two types of discriminant analysis classifiers: linear and quadratic. The difference between them is that linear discriminant creates linear boundaries between classes and the quadratic discriminant creates nonlinear boundaries between classes. In this work, we use a Linear Discriminant classifier because it requires little memory at training, while the quadratic discriminant requires more memory but, in this case, did not give better results.

### 4.2. Performance Metrics

To evaluate the performance of the classifiers, we compute the accuracy, precision, and recall. Validation accuracy is computed as:

accuracy=1−kfoldLoss

where kfoldLoss is the classification error using fivefold cross validation. Precision represents the percentage of the classified items that are relevant results and is computed as:

$$\mathrm{Precision}=\frac{\mathrm{TP}}{\mathrm{TP}+\mathrm{FP}}$$

The recall represents the percentage of the relevant results that are correctly classified and is computed as:

$$\mathrm{Recall}=\frac{\mathrm{TP}}{\mathrm{TP}+\mathrm{FN}},$$

where TP is true positive, FP is false negative, TN is true negative, and FP is false positive.

For each classifier, we use 7 sizes for training sets, starting from 20% of images in the training (while the rest of 80% of images used for testing) and then increasing the percentage of the images used for training up to 80% and decreasing the percentage of images used for testing until 20% is reached. This was performed using an increment step of 10%.

### 4.3. Results for 100 Randomly Selected Rows from Each Image

**Figure 11:**
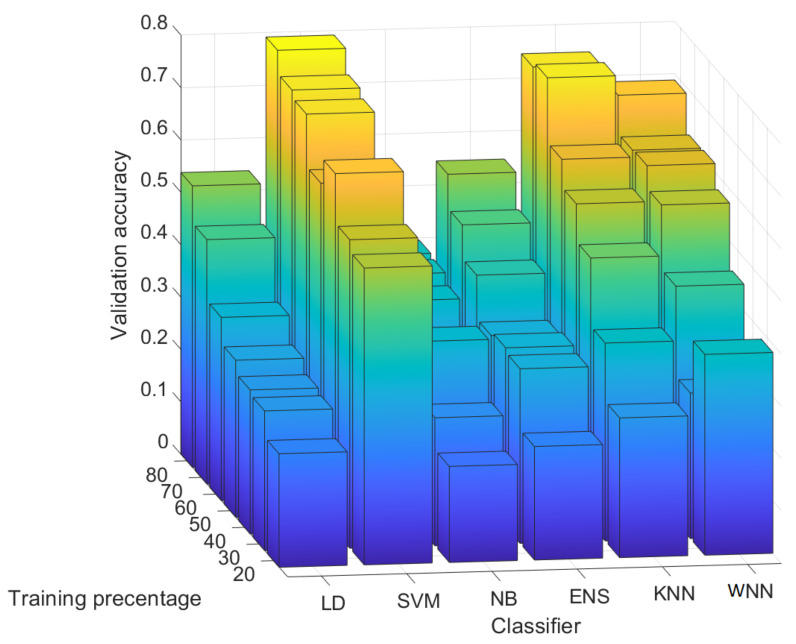
Validation accuracy for 100 randomly selected rows.

In Figure 11, we depict the validation accuracy for all six classifiers and all test scenarios with 100 randomly selected rows for each image. The results from this figure are given as average values for each classifier over all the sensors from our experiments. We also detail in this section the precision and recall for each sensor since the results are not uniform. As expected, for each classifier, the validation accuracy is increasing with the percentage of training data. SVM has the highest validation accuracy for all training percentages followed by the KNN and the WNN. The worst validation accuracy was obtained with NB.

**Figure 12:**
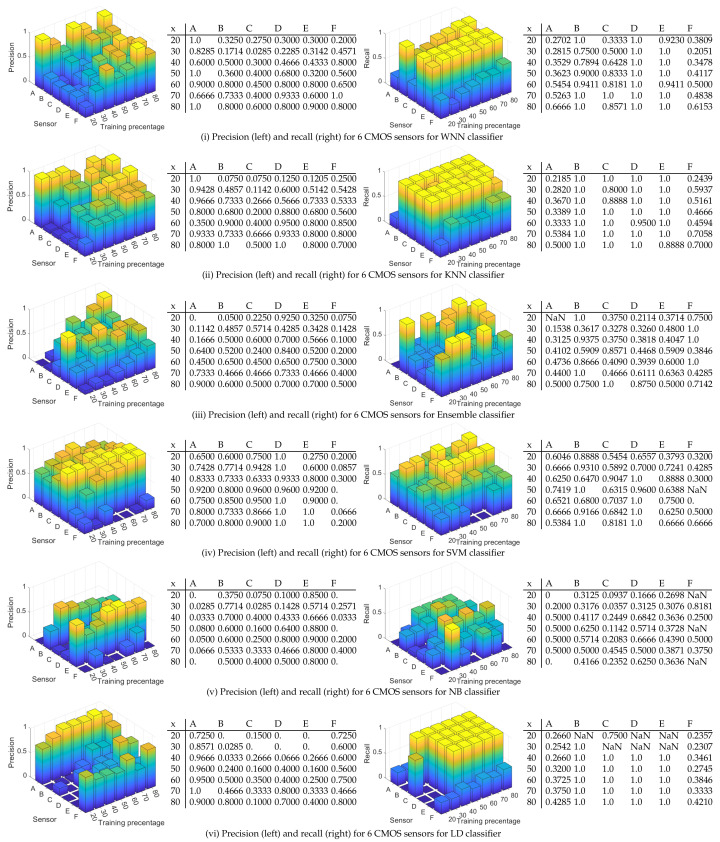
Precision (**left**) and recall (**right**) for 6 CMOS sensors for all tested classifiers for 100 randomly selected matrices.

To give a more accurate depiction in Figure 12, we describe as 3D bar charts and numeric values the precision (left) and the recall (right) for each CMOS sensor for all the tested scenarios and all classifiers in case of 100 randomly selected matrices. Details on these are discussed next:

(i) WNN: For a training percentage below 60%, the values for the precision are below 50% for most of sensors, while for a training percentage of 80%, the lowest precision is 60% for sensors C, 80% for sensors B, D, and F, 90% for sensor E, and 100% for sensor A. In terms of recall, sensor D reached the recall 100% regardless of training percentage. The recall of sensor E is close to 100% for all training percentages. The worst recall was obtained for sensors A and F, but in this case, the results are generally increasing with the training percentage until reaching 66% for sensor A at 80% training and 61% for sensor F.
(ii) KNN: The precision is similar with the results obtained with WNN. For a training percentage below 60%, the results for precision are poor, while for a training percentage of 70%, the lowest precision is increasing to 66% for sensor C. For recall, sensor B is around 100% regardless of training percentage. Sensors C, D, and E reach a recall close to 100% for all training percentages, while sensors A and F have a recall below 70%.
(iii) ENS: The precision and recall are lower that in case of KNN and WNN for all tested training percentages. In case of 20% training for sensor A, the recall value is marked with NaN since the result was not a real number due to a division by zero (there were no true positives and false negatives).
(iv) SVM: For all training percentages, the precision is highest, even close to 100% in many cases for sensors A, B, C, D, and E, while sensor F reaches the maximum value of 30% at 40% training, which is not good. Regarding the recall, for all training percentages, the recall is above 53% for sensors A, B, C, and D, which proves an average performance. At higher training set sizes, sensor E has a recall of around 60–70%, which is again average, and sensor F is between 0–66%, which is not good at all.
(v) NB: The precision and recall are worse than for the rest of the classifiers. For all training percentages, sensors A–F have a precision between 0–90%, which is a mixed result, only for sensor E to be more consistent at around 80%. For all sensors and all training percentages, the recall is below 81%, generally staying at around 30–50%, which is again not good.
(vi) LD: For all training percentages, sensors B, C, D, and E generally have a precision below 50%, which is not good (with a few exceptions), while sensor A and F have a higher precision between 46% and 100%. Concerning the recall, the situation, however, reverses, and sensors A and F, which had better precision now have the worse recall at around 23–42%. While sensors B to E have a recall of 100%, this seems of little use as long as most of the samples for A and F are rejected.

To sum up, the results obtained for 100 randomly selected matrices, sensors B, C, D, and E are identified more easily than sensors A and F with NN, KNN, and LD. The results are still not great, with recalls of 60% or worse for many devices and classifiers, which suggests that we have to increase the size of the feature vectors, which is done next.

### 4.4. Results for 1000 Randomly Selected Rows from Each Image

**Figure 13:**
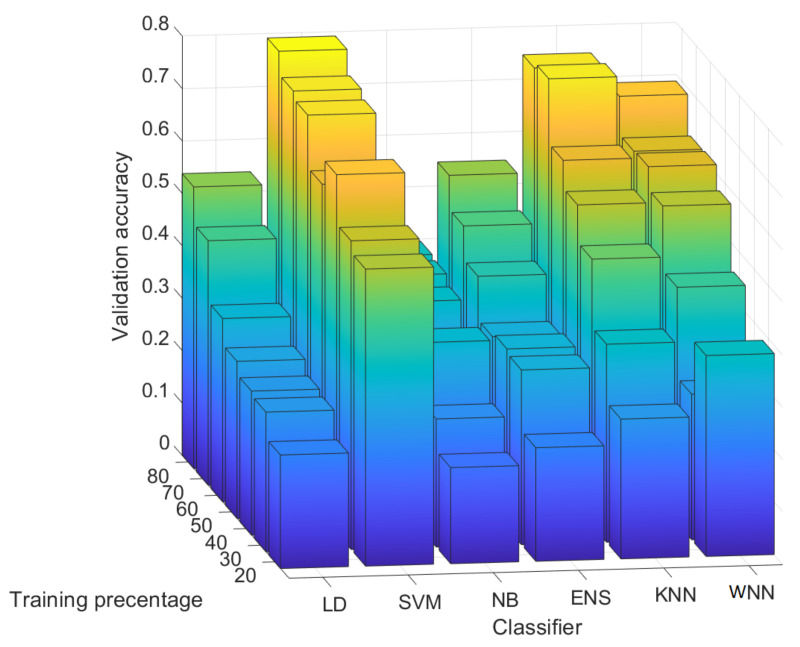
Validation accuracy for 100 randomly selected rows.

**Figure 14:**
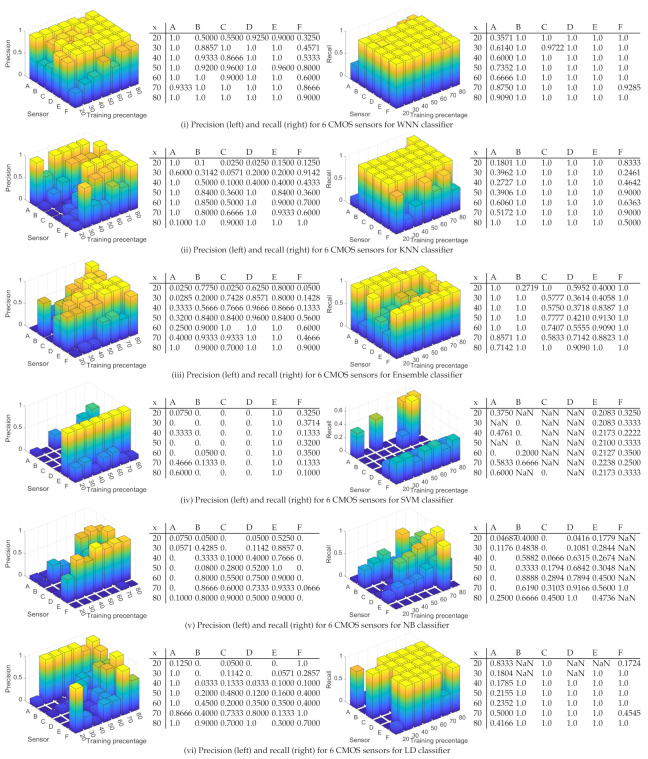
Precision (**left**) and recall (**right**) for 6 CMOS sensors for all tested classifiers for 1000 randomly selected matrices.

In Figure 13, we depict the validation accuracy for all six classifiers and all test scenarios with 1000 randomly selected rows from each image. Again, the results from this figure are given as average values, and we detail the precision and recall for each sensor in what follows since the results are not uniform. As expected, for each classifier, the validation accuracy is generally increasing with the percentage of training data. As can be easily observed, now WNN has the highest validation accuracy for all training percentages followed by the KNN and ENS. The worst validation accuracy was obtained with SVM. In Figure 14, we describe as 3D bar charts and numeric values the precision (left) and recall (right). Again, we discuss in detail the precision and recall on each of the sensors and classifiers:

(i) WNN: For a training percentage above 30%, sensors A–E are identified with a precision above 86%, which is good, while sensor F is identified with a precision between 45% and 90%. For 80% training, sensors A–F are identified with a precision between 90% and 100%, which is very good. Concerning the recall, for all training percentages, the recall for sensors B–F is close or equal to 100%. For sensor A, the recall increases with the training percentage from 35% at 20% training to 90% at 80% training. These results seem satisfactory for all sensors.
(ii) KNN: The results are poorer than the ones obtained with the WNN. For a training percentage below 60%, the precision is poor, while for a training percentage of 60–70%, the precision ranges from 50% to 100%. For 80% training, sensor A has only 10% precision, which is very bad, sensor C has 90% precision, and sensors B, D, E, and F reach a precision of 100%. In terms of recall, for all training percentages, the recall for sensors B–E is 100%, while sensors A and F have a mixed recall between 18% and 100%. Overall, the results with the KNN are not bad, but they are not very stable, e.g., for sensor A, the precision dropped from 100% to 10%.
(iii) ENS: The precision is comparable with the precision obtained in case of the KNN. For a training percentage below 70%, the precision is lowest for sensors A and F, while for sensors B, C, D, and E, it reaches 100%. For 80% training, the precision is 100% for sensors A, D, and E and 90% for B and F, while for C, it is only 70%. For the recall, on sensor F, the recall is 100% for all training percentages and the same 100% is reached for sensor B (except in the case of 20% training, which does not seem enough). For A, C, D, and E, at 80% training, the recall is between 71–100%; below this, the results are poor for D and E, reaching under 50%.
(iv) SVM: For all training percentages, the precision for sensors C and D is zero, which immediately discards this classifier. Sensors A and B similarly lead to 0% precision in some training sets, while sensor F has the precision below 35%. Even if sensor E reaches 100% precision, this is because all sensors are wrongly identified as sensor E. Finally, all sensors have a recall below 66%, which is not good.
(v) NB: The results are comparable with the results in case of SVM. For all training percentages, sensors A and F are identified with a precision close to 0%, while sensors B–E are identified with a precision between 0% and 93%. In terms of recall, the values are generally between 0% and 60%, with a few exceptions, which is not great.
(vi) LD: For all training percentages, sensors B–F generally give a precision below 40%, which is not good, while sensor A reaches 100% precision in most circumstances. Concerning the recall, the situation, however, reverses, and sensor A, which had better precision, now has the worse recall, generally around 20% with some exceptions. While sensors B–F have a recall of 100%, this seems of little use as long as most of the samples for A are rejected, and the precision for B–F was not great either.

To sum up the results for 1000 randomly selected matrices, with the WNN, we obtained by far the best results, followed by KNN at a significant distance. With ENS, the classification still works for high training percentages, while SVM and NB do not work anymore for 1000 randomly selected matrices. The sensors that gave the worst results are sensors A and F. Even for these two, with the WNN, the precision is 90–100%, while the recall is 90.9–100% at 80% training. This proves that the WNN is capable enough to distinguish between the sensors.

*Further discussions on the results.* For 100 rows, the SVM has the highest validation accuracy, followed by KNN and then WNN. ENS and LD have the similar results, while NB gives worst results. Even if SVM has the highest accuracy, only five of the six cameras are correctly identified. This suggests that 100 rows are not enough for identification. In the case that data are insufficient, traditional machine learning algorithms may perform better than neural networks. In the case of 1000 rows, with WNN, we obtained the best results for all training percentages, followed by KNN and then ENS. SVM gives the worst results, even compared to NB in this case. The WNN clearly outperforms traditional machine learning algorithms in the case of 1000 samples.

We ran all classifiers for 10 times, selecting each time other random rows, but the results remained similar. The poor performance of the traditional machine learning algorithms in case of 1000 rows may have been caused by overfitting. In a distinct context, the authors in [70,71] also reported performance degradation for SVM due to overfitting. This may explain why SVM had the best accuracy for 100 rows, and for 1000 rows, it became the worst classifier. We also tried to use PCA (principal component analysis) to optimize the results, but no improvements were seen. In terms of the training time, NB had the highest requirements, up to 60 min for 1000 matrices. Other classifiers required around 1 min in case of 100 rows and below 8 min for 1000 rows.

**Figure 15:**
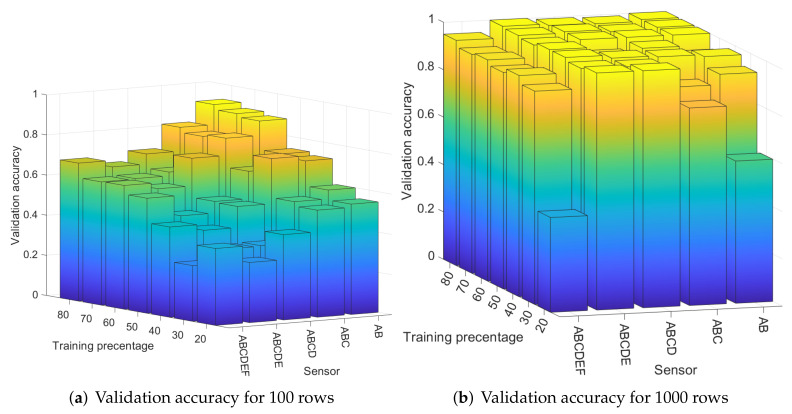
Validation accuracy for 2, 3, 4, 5, and 6 sensors for 100 randomly selected rows (**a**) and 1000 randomly selected rows (**b**) in the case of the WNN classifier.

In order to compare the validation accuracy for classifying a distinct number of sensors, we ran the WNN classifier for two, three, four, and five sensors. In Figure 15, we show the results for 20% to 80% training percentage when two sensors (A and B), three sensors (A, B, and C), four sensors (A, B, C, and D), five sensors (A, B, C, D, and E), and finally all six sensors are classified (A, B, C, D, E, and F). In Figure 15a, we depict the results for 100 rows, and in Figure 15b, we depict the results for 1000 rows. The validation accuracy decreases as more sensors are added, which is visible especially for the case of 100 rows in Figure 15a. When we increase the number of the rows to 1000, the difference in the validation accuracy for classifying two, three, four, five, and six sensors is not significant, as can be seen in Figure 15b. This way, we can confirm that although by increasing the number of sensors the accuracy lowers, the results will still be good if sufficient data is added, e.g., 1000 rows with AC coefficients are used.

## 5. Conclusions

Our work explored smartphone fingerprinting based on the low and mid frequency AC coefficients from the DCT of dark images. The analysis showed the blue channel to be more efficient for identifying the camera. Taking dark images requires a simple action by which users take a picture by keeping the phone in their palm. We used six machine learning algorithms to identify the smartphones. The wide neural network (WNN) gave the best results with an accuracy of 97% for 1000 samples and around 70% for 100 samples. The traditional KNN algorithm also gave good results, reaching around 80% accuracy for both 100 and 1000 samples. The results were obtained for 50 images acquired with six identical cameras from Samsung Galaxy J5 phones. As future work, we may extend the procedures in this paper to a larger pool of sensors and possibly more demanding neural network architectures.

## Figures and Tables

**Table 1:** Overview of various works in which fingerprinting smartphones based on their camera was proposed (the results are for distinct datasets).

Work	Feature	Classifier	Max. Accuracy
[12]	highpass filter	CNN, AlexNet, and GoogleNet	94.5%
[13]	DCT + PCA	RF based ENS	99.1%
[14]	Prb. Repr. and thresholding	SVM	87.6
[15]	social network	ResNet50	96%
[11]	split image	CNN	100%
[16]	Supervised pipeline (rich features, CFA features, and CNN-derived features)	PISVM, ET, and SSVM classifier and CNN	98.68%
[17]	PRNU and noiseprint	CNN + SVM, LRT, and r-LRT	95.5%
[18]	PRNU	CNN	80%
**This work**	DSNU	WNN, KNN, ENS, SVM, NB, and LD	97%

## Data Availability

The sample images from the experiments can be made available on request to the authors.
